# XB130 Mediates Cancer Cell Proliferation and Survival through Multiple Signaling Events Downstream of Akt

**DOI:** 10.1371/journal.pone.0043646

**Published:** 2012-08-23

**Authors:** Atsushi Shiozaki, Grace Shen-Tu, Xiaohui Bai, Daisuke Iitaka, Valentina De Falco, Massimo Santoro, Shaf Keshavjee, Mingyao Liu

**Affiliations:** 1 Latner Thoracic Surgery Research Laboratories, University Health Network Toronto General Research Institute, Toronto, Ontario, Canada; 2 Department of Surgery, Faculty of Medicine, University of Toronto, Toronto, Ontario, Canada; 3 Dipartimento di Biologia e Patologia Cellulare e Molecolare, Istituto di Endocrinologia ed Oncologia Sperimentale del CNR ‘G. Salvatore’, Naples, Italy; Hungarian Academy of Sciences, Hungary

## Abstract

XB130, a novel adaptor protein, mediates RET/PTC chromosome rearrangement-related thyroid cancer cell proliferation and survival through phosphatidyl-inositol-3-kinase (PI3K)/Akt pathway. Recently, XB130 was found in different cancer cells in the absence of RET/PTC. To determine whether RET/PTC is required of XB130-related cancer cell proliferation and survival, WRO thyroid cancer cells (with RET/PTC mutation) and A549 lung cancer cells (without RET/PTC) were treated with XB130 siRNA, and multiple Akt down-stream signals were examined. Knocking-down of XB130 inhibited G_1_-S phase progression, and induced spontaneous apoptosis and enhanced intrinsic and extrinsic apoptotic stimulus-induced cell death. Knocking-down of XB130 reduced phosphorylation of p21Cip1/WAF1, p27Kip1, FOXO3a and GSK3β, increased p21Cip1/WAF1protein levels and cleavages of caspase-8 and-9. However, the phosphorylation of FOXO1 and the protein levels of p53 were not affected by XB130 siRNA. We also found XB130 can be phosphorylated by multiple protein tyrosine kinases. These results indicate that XB130 is a substrate of multiple protein tyrosine kinases, and it can regulate cell proliferation and survival through modulating selected down-stream signals of PI3K/Akt pathway. XB130 could be involved in growth and survival of different cancer cells.

## Introduction

XB130 is a newly discovered adaptor protein for intracellular signal transduction; it is involved in gene regulation, cell proliferation, cell survival, cell migration, and tumorigenesis [1]. Human *xb130* gene was discovered during the cloning process of human *afap* gene [2], [3], [4]. It encodes 818 amino acids with an apparent molecular size of approximately 130 kDa. The overall structure of XB130 shares similarity with AFAP thus is also known as AFAP1 like protein 2 (AFAP1L2). As an adaptor protein, the N-terminal region of XB130 includes several tyrosine phosphorylation sites and proline-rich sequence, which can potentially interact with SH2 and SH3 domain-containing proteins, respectively. The middle portion harbors two pleckstrin-homology (PH) domains that may target proteins to cellular membranes through interactions with specific phospholipids. The C-terminal region contains a coiled-coil domain, which might be involved in protein oligomerization and DNA binding [4]. XB130 can interact and activate c-Src tyrosine kinase, leading to elevated tyrosine phosphorylation of multiple proteins including XB130, and transactivation of AP-1 and SRE [4]. During cell migration, XB130 regulates actin cytoskeleton rearrangement as demonstrated by its translocation to cell periphery in lamellipodia. Silencing endogenous XB130 can cause a decrease in the rate of wound closure, inhibit matrigel invasion, and reduce lamellipodial persistence and cell spreading, which suggest that it plays an important role in cell motility and invasion [5].

XB130 is involved in thyroid cancer cell proliferation and survival [1]. Thyroid cancer is a type of endocrine malignancy, which involves multiple genetic and epigenetic alterations leading to MAPK and PI3K/AKT signaling pathway activation [6], [7], [8]. A common mutation found in thyroid cancer is RET/PTC chromosomal rearrangements. The product RET/PTC is a protein produced from the chromosomal rearrangement with the combination of the 3′ portion of the RET gene and the 5′ section of a partner gene. This results in a constitutively activated tyrosine kinase, RET/PTC [9]. RET/PTC exhibits transforming ability via effecting differentiation, mitogenic and metastatic potential in thyroid cancer [10], [11]. RET/PTC causes a robust tyrosine phosphorylation of XB130, which promotes its association with the p85α subunit of phosphatidylinositol 3-kinase (PI3K). This in turn activates Akt. Down-regulation of XB130 in TPC1 papillary thyroid cancer cells, harboring the RET/PTC kinase, strongly reduced Akt activity, cell-cycle progression and cell survival [12]. Furthermore, in WRO cells, another thyroid cancer cell line with RET/PTC mutation [13], cells stably transfected with XB130 shRNA reduced tumor growth in nude mice. Microarray study identified that multiple genes regulated by XB130 are related to cell proliferation or survival, including many transcription regulators [14].

Since XB130 is highly expressed in thyroid, it has been speculated to be a thyroid-specific tyrosine kinase substrate. Recently, we have found expression of XB130 in esophageal cancer [15], and in other cancer cell lines. In the present study we sought to determine whether XB130 plays a role in cancer cells independent from the presence of RET/PTC. Furthermore, although the PI3K/AKT pathway has been identified as important for XB130-mediated cell proliferation and survival, the downstream signals of Akt are as yet undetermined. Thus, we studied these events with WRO cells, a human thyroid cancer cell line with RET/PTC rearrangement, and A549, a human lung adenocarcinoma cell line without RET/PTC.

## Materials and Methods

### Cell Lines, Antibodies and Other Reagents

Human follicular thyroid carcinoma WRO cells (established by Dr GJF Juillard, University of California-Los Angeles School of Medicine, Los Angeles, CA, USA) were maintained in RPMI 1640, supplemented with 10% FBS, 1 mM pyruvate and non-essential amino acids (GIBCO-BRL, Gaithersburg, MD, USA) [13]. Human lung adenocarcinoma A549 cells, obtained from ATCC (CCL-185; Manassas, VA) [16], were grown in DMEM medium, supplemented with 10% FBS, 1% penicillin-streptomycin, and 1% glutamine. Cells were cultured in a standard humidified incubator at 37°C with 5% CO_2_.

Expression vectors for RET/PTC3, activated versions of ABL and SRC [17], or EGFR and ERBB2 [18], which are activated upon overexpression, are described elsewhere. Monoclonal XB130 antibody was generated as described previously [4]. Antibodies for phospho-Akt (Ser473), Akt, phospho-GSK-3β (Ser9), p21Cip1/WAF1, p27Kip1, p53, phospho-FoxO3a (Thr32), FoxO3a, phospho-SAPK/JNK (Thr183/Tyr185), SAPK/JNK, phospho-p38 MAPK (Thr180/Tyr182), p38 MAPK, phospho-p44/42 MAPK (Thr202/Try204), phosho-Src (Try416) were from Cell Signaling Technology (Beverly, MA, USA); monoclonal anti-phosphotyrosine antibodies were from Upstate Biotechnology Inc. (Lake Placid, NY, USA) and anti-c-myc tag (sc-40) were from Santa Cruz Biotechnology (Santa Cruz, CA, USA). Antibodies for GAPDH, ERK1, phospho-p21 (Thr145), caspase-8 and caspase-9 were from Santa Cruz Biotechnology (Santa Cruz, CA, USA). Anti-PCNA antibody was from Abcam (Cambridge, MA, USA). Anti-Ki-67, clone Ki-S5, was from Chemicon (Temecula, CA, USA). Anti-Src (GD11) and anti-Fas mAb (clone CH11) were from Upstate Biotechnology Inc. (Lake Placid, NY, USA). Anti-Phospho-p27Kip1 (Thr157) antibody was from R&D Systems Inc. (Minneapolis, MN, USA). Anti-p85α of PI3K antibody was from BD PharMingen (San Diego, CA, USA). Staurosporine was from Sigma-Aldrich (St. Louis, MO, USA).

### Protein Studies

Immunoblotting experiments were performed according to standard procedures described previously [2], [3]. Briefly, Cells were lysed with modified radioimmune precipitation assay buffer (50 mM Tris-HCl; pH 7.5, 150 mM NaCl, 2 mM EGTA, 2 mM EDTA and 1% Triton X-100) containing 10 µg/ml each aprotinin, leupeptin, pepstatin, 1 mM phenylmethylsulfonyl fluoride, 1 mM Na_3_VO_4_, and 10 mM NaF. Protein concentration was measured with a modified Bradford assay (Bio-Rad, Munich, Germany). Cell lysates containing equal amount of total proteins were separated by SDS-PAGE and then transferred onto nitrocellulose membranes (Schleicher & Schuell, Whatman, Middlesex, UK). Membranes were then probed with the indicated antibodies. Proteins were revealed by an enhanced chemiluminescence detection kit (ECL) (Amersham Pharmacia Biotech, Little Chalfort, UK). Band densities were quantified using the ImageJ software (http://rsb.info.nih.gov/ij/) after being scanned from the film. The protocol for immunoprecipitation has been described previously in detail [3], [19].

### siRNA Transfection

Two siRNAs were designed according to XB130 sequence as described previously [4], [12]. Cells were transfected with 50 nM pooled XB130 siRNAs using the oligofectamine reagent (Invitrogen, San Diego, CA, USA) according to standard procedures described previously [4], [20]. The medium containing siRNA was replaced with fresh medium after 24 h. The siSTABLE V2 non-targeting siRNA#1 from Dharmacon (Lafayette, CO, USA) was used as a negative control. For protein studies, siRNA transfected cells were harvested at 48 h after transfection.

### Real-time Quantitative RT-PCR

Sequence-specific primers for human RET/PTC were designed by Balogh et al.: 5′-GTCGGGGGGCATTGTCATCT-3′ and 5′-AAGTTCTTCCGAGGGAATTC-3′ [21]. Total RNA was extracted using RNeasy kit (Qiagen, Valencia, CA), and cDNA was synthesized from total RNA using MuLV Reverse Transcriptase (Applied Biosystems, Carlsbad, CA). Quantitative RT-PCR was performed using SYBR Green I Master PCR kit on LightCycler480 (Roche, Indianapolis, IN). Each assay included a standard curve of five serial dilutions and a no-template negative control. All assays were performed in triplicate. Gene expression levels were normalized to the level of SDHA, a housekeeping gene [14].

### Cell Cycle Analysis

Cell cycle distribution was analyzed by flow cytometry [22]. Briefly, cells were harvested in phospho-buffered saline (PBS), and fixed in 70% cold ethanol overnight. Fixed cells were washed twice in PBS and permeabilized with 0.1% Triton X-100 and 2 mg/ml RNase A in PBS for 30 min. They were then washed once in PBS and stained with 50 µg/ml of propidium iodide (PI) (Sigma-Aldrich). Stained cells were analyzed with the Cytomix FC500 flow cytometry system (Beckman-coulter, Fullerton, CA, USA).

### Analysis of Apoptotic Cells

After treated with staurosporine or FasAb for 24 h, cells were harvested and stained with flourescein isothiocyanate (FITC)-conjugated Annexin V and PI using the Annexin V kit (Beckman Coulter, Brea, CA, USA) following manufacturer’s protocols and analyzed by the Cytomix FC500 flow cytometry system.

### Statistical Analysis

Statistical analysis was carried out using Student’s t-test. Differences were considered significant when the P value was less than 0.05. Statistical analyses were performed using the statistical software JMP version 5 (SAS Institute Inc., Cary, NC, USA).

## Results

### XB130 Controls Cell Cycle Progression of Cancer Cells

**Figure 1:**
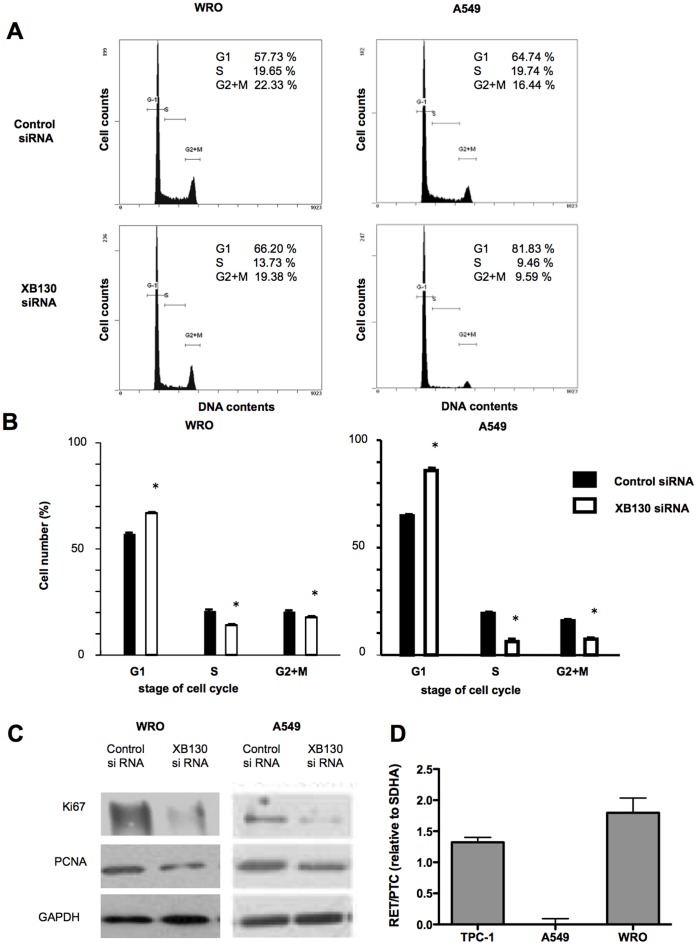
XB130 controls cell cycle progression of cancer cells. (A and B) Down-regulation of XB130 inhibited G_1_-S phase progression in WRO and A549 cells. Cells transfected with control or XB130 siRNA were stained with PI and analyzed by flow cytometry. Mean ± SEM. *n* = 6. *p<0.05 (compared with control siRNA). (C) Reduced Ki67 and PCNA levels in XB130 siRNA treated WRO and A549 cells, as determined by western blotting. *n* = 3. *p<0.05 (compared with control siRNA treated group). (D) Presence of RET/PTC rearrangement was confirmed in both TPC-1 and WRO cells using RT-PCR. A549 cells do not contain RET/PTC rearrangement.

To explore the role of XB130 in cancer cell cycle progression we conducted knockdown experiments using XB130 siRNA in human thyroid follicular carcinoma WRO cells (with RET-PTC rearrangement) and in human lung adenocarcinoma A549 cells (a cell line commonly used in lung cancer studies [15]). Two siRNAs targeting different sites of XB130 were designed. We have previously shown that both of them effectively reduced XB130 protein levels and Akt phosphorylation in TPC-1 cells [12] and other cell types (data not shown). Combined application of these two siRNAs reduces the concentration of each siRNA to half, thus may reduce potential off-target effects. Therefore, we have used this strategy previously [5], [12] and also in the present studies. Flow cytometry showed that down-regulation of XB130 partially blocked cell cycle progression from G_1_ to S phase in both WRO and A549 cells as determined by flow cytometry (Fig. 1A). There are significantly more cells in G1 phases than in either the S or G2/M phase (Fig. 1B). While, western blots illustrate a significant reduction in cell proliferation markers, Ki-67 and PCNA, in the XB130 siRNA treated cells (Fig. 1C). The presence of RET-PTC rearrangement was confirmed in TPC-1 cells (as a positive control) and in WRO cells, whereas it is absence in A549 cells as shown by RT-PCR (Fig. 1D). These results indicate that XB130 plays important roles in the regulation of cancer cell proliferation either in the presence or the absence of RET/PTC.

### XB130 Controls Survival of Cancer Cells

**Figure 2:**
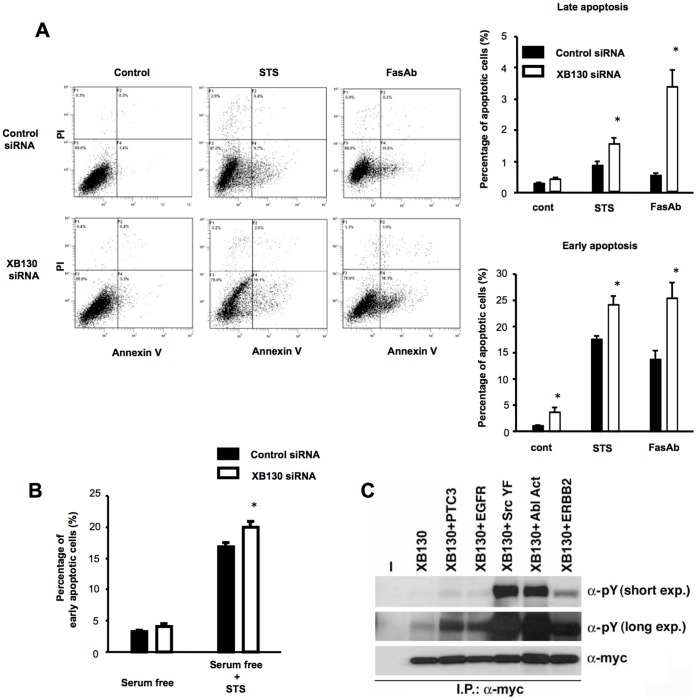
XB130 controls cell survival of cancer cells. (A) Down-regulation of XB130 enhanced spontaneous and induced cell death of WRO cells cultured with 10% FBS. Cells transfected with control or XB130 siRNA were treated with staurosporine (STS, 200 nM), or Fas antibody (FasAb, clone CH11, 100 ng/ml) for 24 h. Apoptosis was determined by flow cytometry using PI/Annexin V double staining. (B) Down-regulation of XB130 enhanced staurosporine-induced apoptosis in serum free condition in A549 cells. A549 cells transfected with control or XB130 siRNA were incubated in serum free medium with or without 200 nM STS for 24 h. *n* = 6. Mean ± SEM. *p<0.05 (compared with control siRNA). (C) XB130 can be phosphorylated by protein tyrosine kinases other than RET/PTC. Immunoprecipitation with anti-myc antibody in HEK293 cells transfected with myc-tagged XB130, along with RET/PTC3, EGFR, Src, Abl, or ERBB2, showed an increase in XB130 tyrosine phosphorylation using anti-phosphotyrosine antibody.

To determine the role of XB130 in cancer cell survival, we treated WRO and A549 cells with XB130 siRNA and used flow cytometry to quantify and to discriminate between the extrinsic (death receptor mediated) apoptotic pathway, and the intrinsic (mitochondrial mediated) apoptotic pathway [23]. Down-regulation of XB130 induced early apoptosis (Annexin V positive/PI negative) in WRO cells at 48 h after transfection of siRNA (Fig. 2A). Further, XB130 siRNA enhanced extrinsic (FasAb, clone CH11, 100 ng/ml) and intrinsic (staurosporine, 200 nM) apoptotic stimulus-induced early and late apoptosis (Annexin V/PI double positive) (Fig. 2A). On the other hand, in A549 cells cultured in the medium containing 10% FBS, down-regulation of XB130 did not enhance spontaneous nor the staurosporin-or FasAb-induced cell death, even at 500 ng/ml of FasAb (Fig. S1A). Further, combined use of FasAb (500 ng/ml) and IFN-γ (100 ng/ml), a condition that has been previously reported to induced cell death in several other types of cells [24], [25], did not induce apoptosis in A549 cells (Fig. S1B). Western blotting revealed that the expression of endogenous Fas in A549 cells is even higher than that in WRO cells (Fig. S1C). However, under serum free condition, XB130 siRNA treatment enhanced staurosporine-induced early apoptosis in A549 cells (Fig. 2B). These findings indicate that A549 cells are resistant to both extrinsic and intrinsically mediated apoptotic signals. Nevertheless, XB130 is involved in cell survival in both cell lines under divergent experimental conditions.

Even though A549 cells do not have RET/PTC, other endogenous PTKs could phosphorylate XB130. Accordingly, we have shown dramatic protein tyrosine phosphorylation in A549 cells, which can be effectively reduced by SRC inhibitor, PP2 [26]. To test whether whether other protein kinases could phosphorylate XB130 in tyrosine, HEK293 cells, a cell line commonly used to test protein-protein interaction, were transfected with myc-tagged XB130 together with RET/PTC3 as a control, or other membrane-bound (EGFR, ERBB2) or cytosolic (SRC, ABL) tyrosine kinases. Cell lysates were immunoprecipitated with anti-myc antibody and blotted with anti-phosphotyrosine antibody. Though at different levels, all the protein tyrosine kinases co-expressed with XB130 increased its tyrosine phosphorylation (Fig. 2C).

### XB130 Binds to p85α Subunit of PI3K and Controls Akt Activity in Cancer Cells

The two well known signaling cascade that are implicated in the regulation of cell progression and survival via protein phosphorylation are the PI3K/Akt and p38 MAPK pathways [7]. We tested key signaling components in these two pathways and found that the phosphorylation Src, JNK and ERK was not affected in XB130 siRNA treated cells (Fig. S2). Moreover, the phosphorylation of p38 was even significantly increased after XB130 siRAN treatment in WRO cells (Fig. S2), which further exclude the possibility of p38 as a down-stream signal that mediates XB130-related cell proliferation and survival.

**Figure 3:**
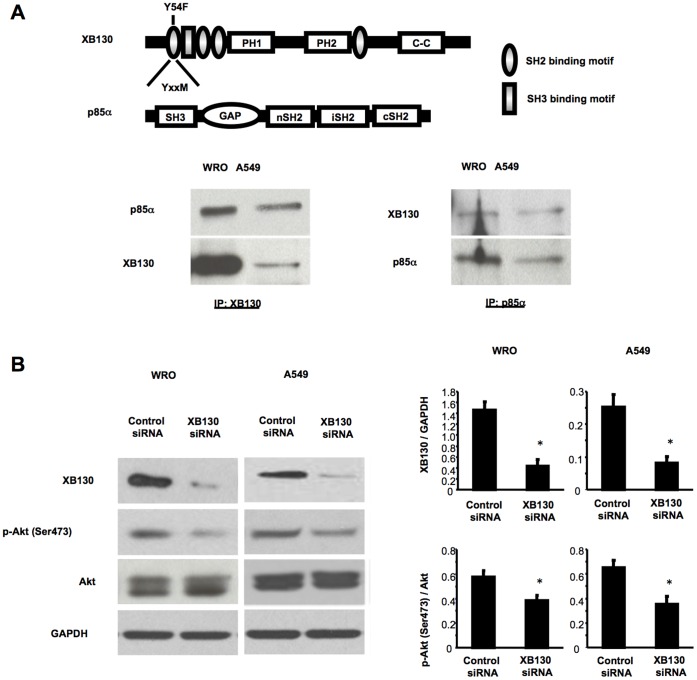
XB130 binds to p85α subunit of PI3K and controls Akt activity in cancer cells. (A) Schematic representation of protein structures of XB130 and p85α subunit of PI3K. Interactions of XB130 with p85α were detected by co-immunoprecipitation in WRO and A549 cells. (B) XB130 siRNA effectively reduced XB130 protein levels and Akt phosphorylation in WRO and A549 cells. siRNA transfected cells were harvested at 48 h after transfection. *n* = 5. *p<0.05 (compared with control siRNA).

We have previously shown that XB130 regulate thyroid cancer cell cycle progression and survival through its interaction with PI3K, leading to the activation of Akt. XB130 has an YxxM motif in the N-terminus starting at the tyrosine 54 that can bind to either the N-or the C-terminal SH2 domain of p85α-subunit of PI3K (Fig. 3A), as demonstrated by GST-fusion protein pull-down assays, co-immunoprecipitation, phosphor-peptide competition, and use of XB130 mutants [12]. Recently, the binding between XB130 and p85 was also reported by Yamanaka et al. in rat thyroid FRTL-5 cells with MOLDI-TOF MS assay [27]. In both WRO and A549 cells, binding between XB130 and p85α subunit of PI3K was shown by co-immunoprecipitation (Fig. 3A). Akt is a downstream target of PI3K, and its phosphorylation at Ser 473 is a sensitive indicator of Akt activity [28]. XB130 siRNA effectively reduced XB130 protein levels; a phenomenon associated with decreased phosphorylation of Akt in both WRO and A549 cells (Fig. 3B).

### XB130 Controls Cancer Cell Cycle Progression and Survival via Multiple Akt Down Stream Molecules

**Figure 4:**
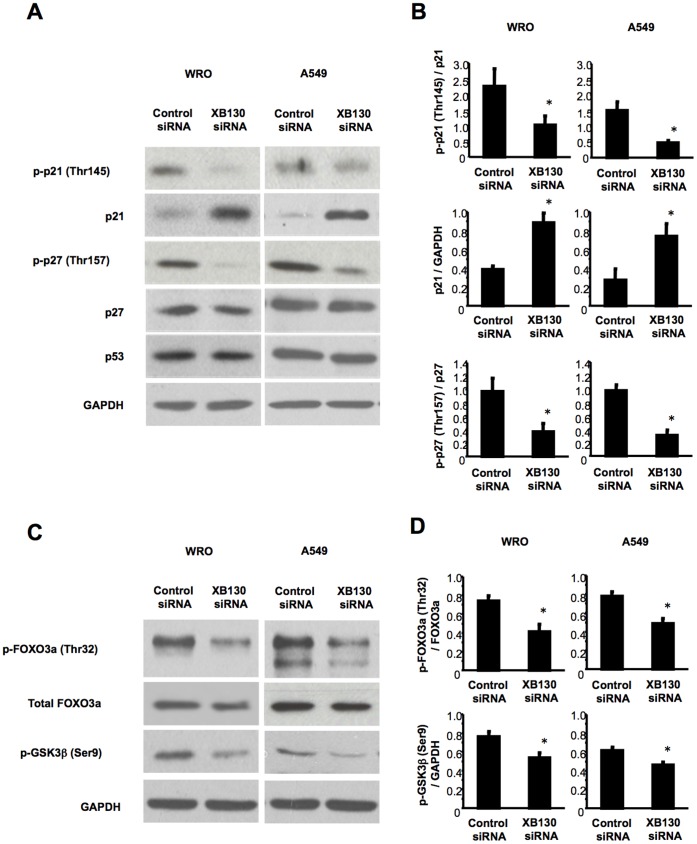
XB130 controls cell cycle progression and survival via PI3K/Akt in cancer cells. (A and B) Down-regulation of XB130 decreased phosphorylation of p21 and p27, and increased expression of p21 in WRO and A549 cells. Expressions of p27 and p53 were not affected by down-regulation of XB130. (C and D) Down-regulation of XB130 decreased phosphorylations of FOXO3a and GSK3β in WRO and A549 cells. *n* = 4. *p<0.05 (compared with control siRNA).

p27Kip1 and p21Cip1/WAF1 are involved in the regulation of cell cycle progression through the inhibition of cyclin-dependent kinases CDK1 and CDK2. The cyclin-dependent kinase inhibitor p27Kip1 nuclear translocation can cause G1 arrest; Akt is able to phosphorylate p27Kip1, and thus block its translocation and function [29], [30], [31]. The cyclin-dependent kinase inhibitor p21Cip1/WAF1 can negatively modulate cell cycle progression by inhibiting the activation of cyclin/cdk2 complex [32]. Activation of Akt can also phosphorylate p21Cip1/WAF1 and keep it in the cytoplasm, which may be critical for cell survival [33]. After XB130 siRNA treatment, the phosphorylation of p27Kip1 and p21Cip1/WAF1 were significantly reduced in both WRO and A549 cells (Fig. 4A and B).

Down-regulation of XB130 also significantly increased the total protein level of p21Cip1/WAF1 (Fig. 4A and B). FOXO3a can bind to and activate the promoter of p21Cip1/WAF1, which has forkhead binding elements adjacent to a SMAD-binding element. PI3K/Akt mediated FOXO3a phosphorylation may lead to its export from the nucleus and subsequently prevent p21Cip1/WAF1 gene expression [34]. The XB130 knockdown reduced the phosphorylation of FOXO3a (Thr32), without affecting the total FOXO3a levels (Fig. 4C and D). This decreased phosphorylation of FOXO3a may help to increase the p21Cip1/WAF1 expression. Among FOXO family members, FOXO1 is known to stimulate transcription of p27Kip1 [35]. The XB130 knockdown was unable to change the phosphorylation of FOXO1 (Thr24) and FOXO1 (Ser256) (data not shown). Coincidently, XB130 siRNA treatment had no effect on p27Kip1 protein levels in both cell lines (Fig. 4A and B).

Another mechanism by which Akt can promote cell proliferation is to phosphorylate and inactivate GSK3β, thus protecting cyclin D1, enhancing CDK4/CDK6 activity, and facilitate cell entry into S phase of the cell cycle [36]. Phosphorylation of GSK3β was decreased after XB130 siRNA treatment (Fig. 4C and D). The tumor suppressor protein p53 is a transcription factor that can induce either growth arrest or apoptosis. Its levels and activities are mainly controlled by MDM2, which can bind p53 directly and promote its ubiquitination and degradation. Akt can phosphorylate MDM2 and thus increase p53 degradation [37]. Down-regulation of XB130 with siRNA, however, did not affect p53 levels.

**Figure 5:**
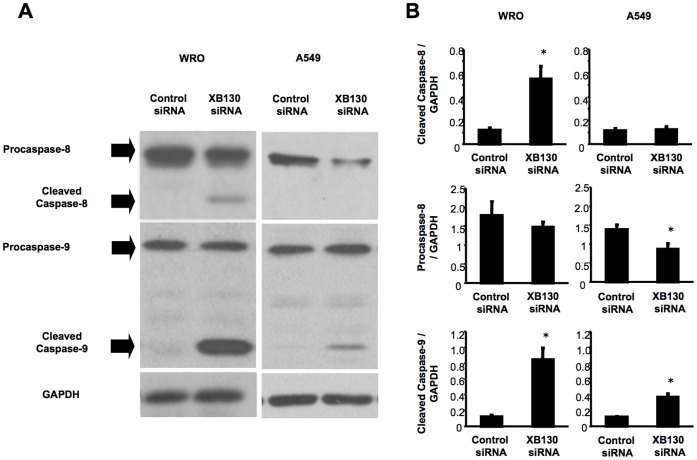
XB130 regulates cell survival through caspase-8 and caspase-9 signaling. (A and B) Cleaved fragments of both caspase-8 and-9 were distinctly increased by knocking-down of XB130 in WRO cells. In A549 cells, down-regulation of XB130 decreased procaspase-8 and increased cleaved caspase-9. *n* = 4. Mean ± SEM. *p<0.05 (compared with control siRNA).

Akt can inhibit apoptosis through multiple mechanisms. For example, Akt is able to phosphorylate procaspase-9, preventing its cleavage into the pro-apoptotic caspase-9, which mediates intrinsic signal initiated apoptosis [38]. Inhibition of Akt enhanced TRAIL-induced activation of caspase-8, which mediates extrinsic signal initiated apoptosis [39]. Down-regulation of XB130 increased the cleavage of caspase-8 and caspease-9 in WRO cells (Fig. 5A and B). In A549 cells, down-regulation of XB130 decreased procaspase-8 level and increased cleaved caspase-9 (Fig. 5A and B). Activation (cleavage) of caspase-8 and caspase-9 are essential steps for extrinsic and intrinsic pathways of cell death, respectively [23]. This may explain why both extrinsic and intrinsic signal induced cell death was enhanced by XB130 siRNA treatment.

Phosphorylation of p21Cip1/WAF1 by Akt may result in its cytoplasmic accumulation and promotes cell survival [40]. Forkhead family transcription factors can trigger apoptosis by inducing the expression of genes that are critical for cell death, such as Bim and Fas ligand [41], [42]. Akt can phosphorylate FOXO3a, to block its translocation from cytoplasm into the nucleus [41]. Thus, down-regulation of XB130 with siRNA reduced the phosphorylation of p21Cip1/WAF1 (Fig. 4A and B) and FOXO3a (Fig. 4C and D). Taken together, these results suggest that XB130 is involved in the regulation of cell proliferation and survival via multiple molecules down-stream of PI3K/Akt pathway.

## Discussion

XB130 has been found to be a substrate and binding partner of RET/PTC, an oncogenic protein tyrosine kinase [12]. Recently, we have found expression of XB130 in a variety of cell lines derived from thyroid, lung, esophageal, pancreatic, and colon cancers. In the present study, we demonstrated that XB130 is involved in proliferation and survival of WRO thyroid cancer cells (with RET/PTC mutation) and A549 lung carcinoma cells (without RET/PTC mutation). Down-regulation of XB130 with siRNA also reduced proliferation and survival of esophageal cancer cells (data not shown). These results indicate that in addition to RET/PTC XB130 may mediate activities of other protein tyrosine kinases. Indeed, when co-expressed with XB130, several receptor tyrosine kinases and non-receptor tyrosine kinases were able to significantly increase phosphorylation of XB130. It has been recently shown that Src family inhibitors, PP1 or PP2, abolished cAMP-induced tyrosine phosphorylation of XB130 and its interaction with p85 PI3K [27]. Therefore, it is plausible that XB130 could be a substrate of multiple protein tyrosine kinases, and it may mediate cell-cycle progress and survival in multiple cancer cell types.

**Figure 6:**
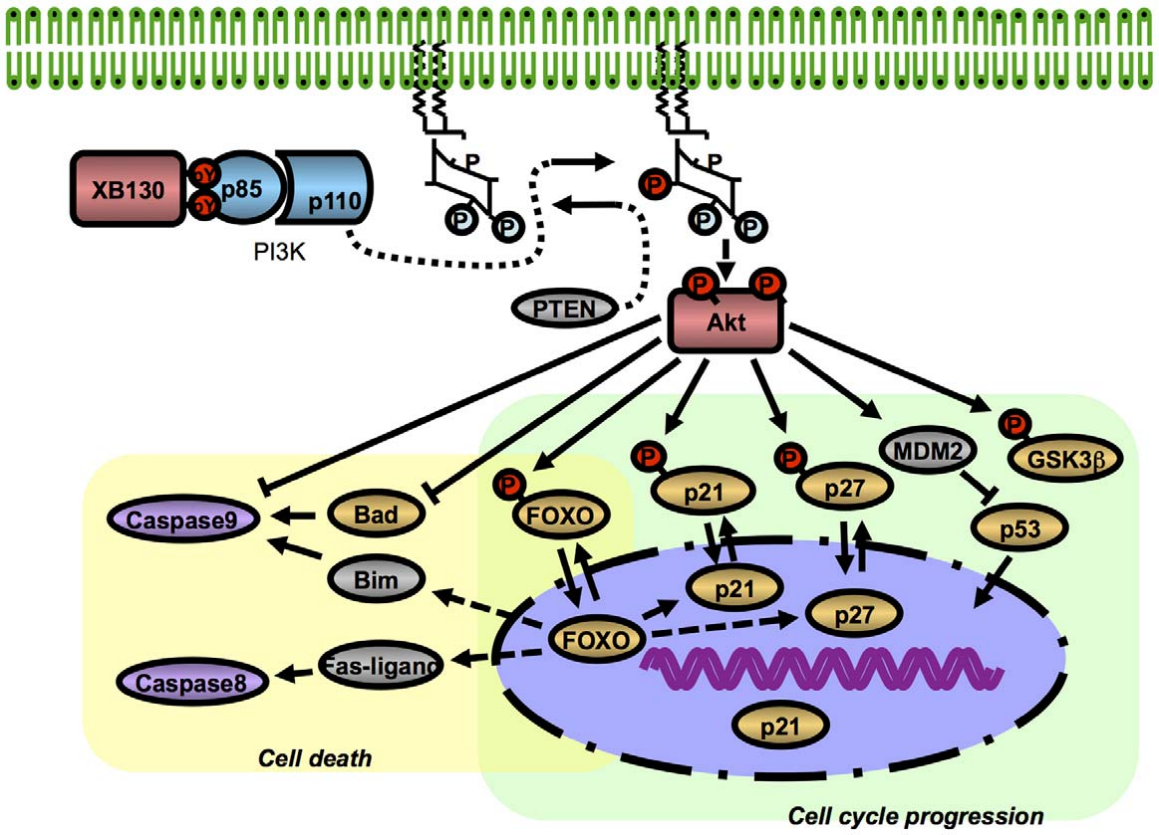
Roles of XB130 in cell cycle progression and survival of cancer. XB130 specifically binds p85α subunit of PI3K, which subsequently activate Akt. Akt plays an essential role in cell proliferation and survival. Down-regulation of XB130 with siRNA affected multiple molecules down-stream of Akt. This suggests that XB130 is an important regulator in PI3K/Akt related cancer cell proliferation and survival.

In the last decade, there has been an increasing focus on the PI3K/Akt pathway as one of the central pathways for cell proliferation and survival [43], [44], [45]. The *class-Ia* PI3Ks are heterodimers with a regulatory subunit (p85α, p85β or p55δ) and a p110 catalytic subunit (p110α, p110β or p110δ) [46]. Specific phosphotyrosine residues on activated growth factor receptors or on adaptor proteins can bind to SH2 domains of p85, which increases the enzymatic activity of the p110 catalytic subunit and also recruits the enzyme to membrane, where it catalyzes the formation of lipid second messenger, PIP3, by phosphorylate phosphoatidylinositol-4,5-bisphosphate (PIP2) at the 3′ position on its inositol ring [47] (Fig. 6). We have found a consensus p85 binding site (YxxM) in the N-terminus of XB130 that can bind to either the N-or the C-terminal SH2 domain of p85α determined by GST-fusion protein pull-down assays, and that XB130 is co-immunoprecipitated with p85α in human thyroid carcinoma TPC-1 cells. Phospho-peptide containing the YxxM sequence of XB130 (but not its none-phosphorylated form) reduced XB130/p85 interaction and deletion of N-terminus abolished interaction between XB130 and p85 [12]. In a recent study, Yamanaka et al. found rat XB130 was associated with p85 subunit of PI3K, and they named it as PI3KAP. This interaction is essential to enhance cAMP-induced amplification of IGF mitogenic activity in FRTL-5 thyroid cells [27]. In the present study, interaction between XB130 and p85 was found in both thyroid and lung cancer cells. Importantly, down-regulation of XB130 affected many down-stream proteins of PI3K/Akt pathway. These evidences indicate that XB130 is an important regulator of PI3K pathway through its specific binding with p85.

Activation of Akt is a feature of thyroid cancers [48], [49], [50]. Lung tumors also show a high level of phosphorylated Akt [51]. Treatment of thyroid or lung cancer cells with PI3K inhibitors, LY294002 or wortmannin, resulted in decreased cell growth or viability [48], [49], [50], [52], [53], [54]. Due to its critical role in regulating gene transcription, cell cycle progression, and survival, Akt has been implicated in many types of cancer [47], [55]. Identification of XB130 as an effective up-stream regulator of PI3K/Akt pathway may reveal new therapeutic targets for cancer therapeutics.

Oncoproteins may enhance tumor growth by altering cell cycle progression and/or modulating cell death. Down regulation of XB130 with siRNA not only reduced cell cycle progression but also enhanced cell death, indicating that XB130 is a key upstream regulator of both cellular processes. Our results indicate that XB130 regulates cell cycle progression and survival through multiple molecules, including p21Cip1/WAF1, p27Kip1, FOXO3a, GSK3β, Caspase 8 and Caspase 9 (Fig. 6). Although we did not examine all known substrates of Akt, our results strongly suggest that XB130 exerts its regulatory function of cell proliferation and survival through the PI3K/Akt pathway. Furthermore, down-regulation of XB130 did not affect down-stream regulators FOXO1 and p53 indicating that in addition to XB130 related Akt activity, these molecules can be further regulated by other signaling mechanisms, which in turn ensures specificity of XB130-related functions.

In summary, we showed that XB130 could regulate cell proliferation and survival through modulating the PI3K/Akt pathway in thyroid and lung cancer cells. XB130 could be a novel oncoprotein in multiple cancers. RNA interference has become a powerful tool to modulate gene function both *in vitro* and *in vivo* [56], [57]. Targeting XB130 expression with this technique could be an attractive option for cancer therapy.

## Supporting Information

Figure S1 **Down-regulation of XB130 with siRNA did not affect apoptosis in A549 cells in the presence of 10% FBS.** A549 cells were cultured in DMEB plus 10% FBS. (A) Down-regulation of XB130 didn’t enhance spontaneous and induced cell death. A549 cells were treated with 200 nM STS, or 500 ng/ml FasAb for 24 h. (B) FasAb (500 ng/ml) with IFN-γ (100 ng/ml) did not induce apoptosis, as analyzed by flow cytometry using PI/Annexin V double staining. *n* = 3. Mean ± SEM. (C) Expression of Fas were confirmed in A549 and WRO cells by western blotting.
(TIFF)

Figure S2 **Phosphorylation levels of Src and MAPKs in WRO and A549 cells transfected with control or XB130 siRNA.** Phosphorylations of Src, ERK and JNK were not affected by down-regulation of XB130 in WRO and A549 cells, whereas phosphrylation of p38 was increase in WRO cells. *n* = 4. Analyses were performed by western blotting. Mean ± SEM. *p<0.05 (compared with control siRNA).
(TIFF)
