# Supplementary figures and images for: XB130 Mediates Cancer Cell Proliferation and Survival through Multiple Signaling Events Downstream of Akt
Source: PLoS One. 2012 Aug 23;7(8):e43646. doi: 10.1371/journal.pone.0043646 (PMC3426539; doi:10.1371/journal.pone.0043646)

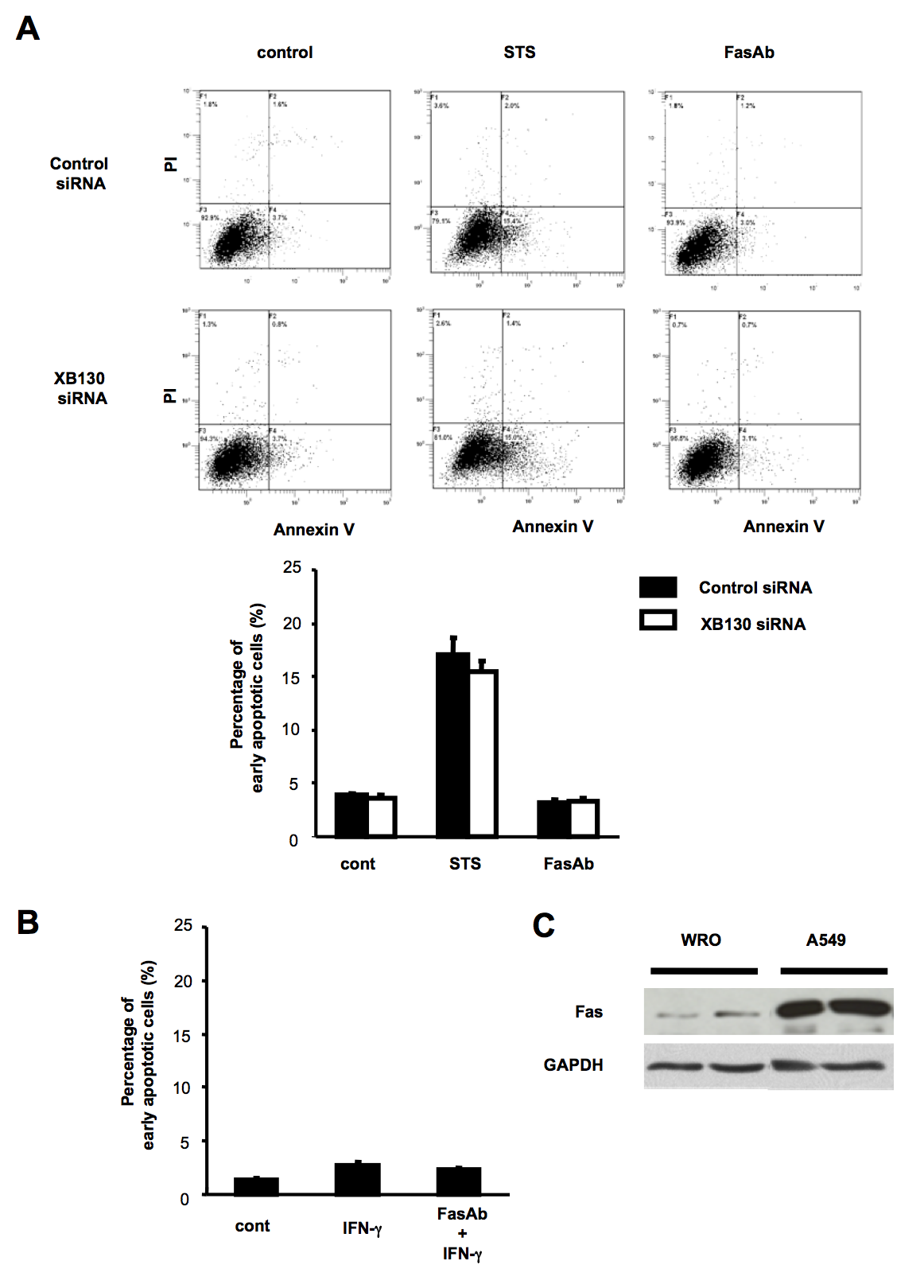

Supplement: Figure S1 — Down-regulation of XB130 with siRNA did not affect apoptosis in A549 cells in the presence of 10% FBS. A549 cells were cultured in DMEB plus 10% FBS. (A) Down-regulation of XB130 didn’t enhance spontaneous and induced cell death. A549 cells were treated with 200 nM STS, or 500 ng/ml FasAb for 24 h. (B) FasAb (500 ng/ml) with IFN-γ (100 ng/ml) did not induce apoptosis, as analyzed by flow cytometry using PI/Annexin V double staining. n = 3. Mean ± SEM. (C) Expression of Fas were confirmed in A549 and WRO cells by western blotting. (TIFF) [file pone.0043646.s001.tiff]

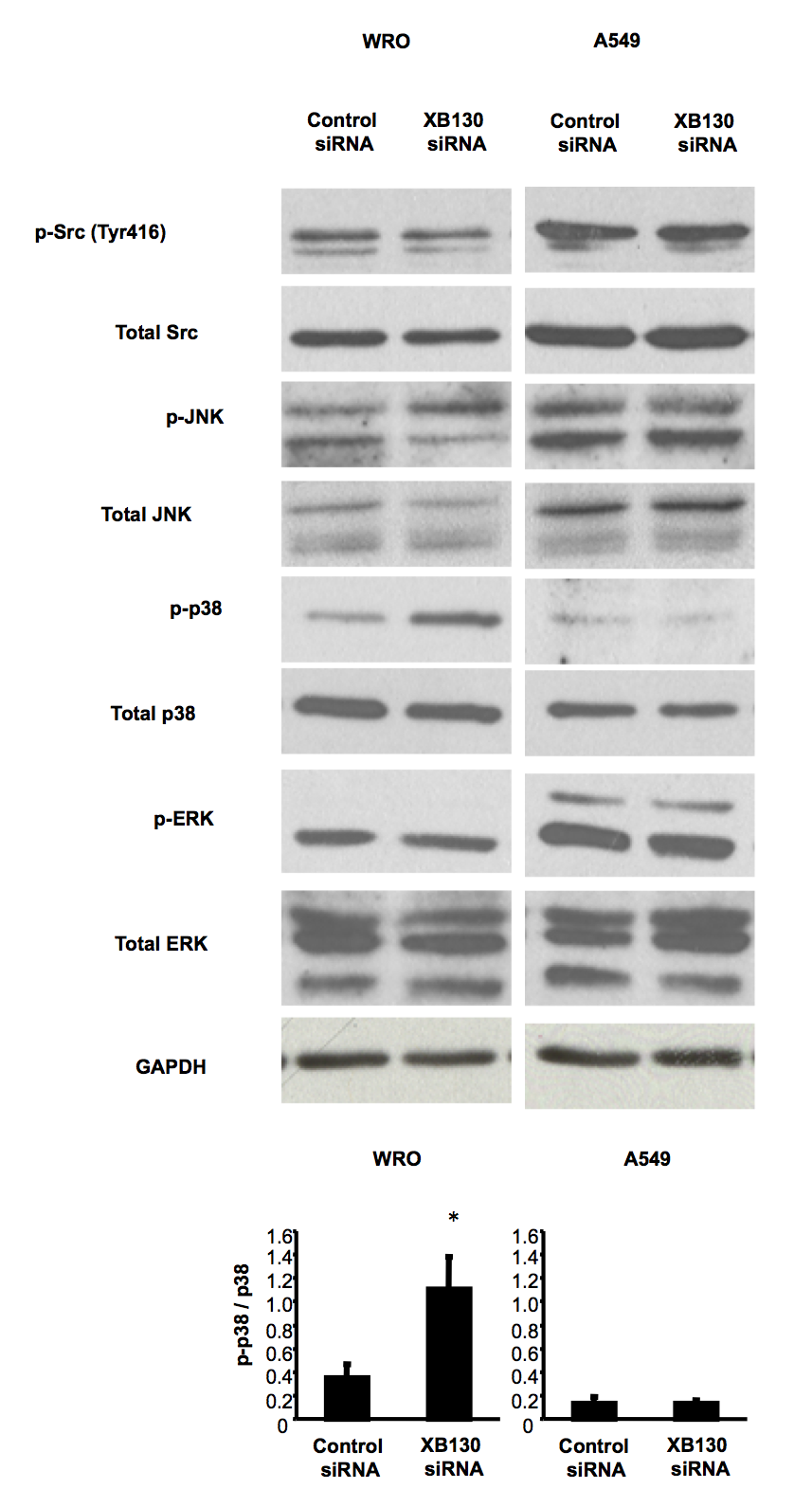

Supplement: Figure S2 — Phosphorylation levels of Src and MAPKs in WRO and A549 cells transfected with control or XB130 siRNA. Phosphorylations of Src, ERK and JNK were not affected by down-regulation of XB130 in WRO and A549 cells, whereas phosphrylation of p38 was increase in WRO cells. n = 4. Analyses were performed by western blotting. Mean ± SEM. *p<0.05 (compared with control siRNA). (TIFF) [file pone.0043646.s002.tiff]
